# Polymorphisms in *IL12A *and cockroach allergy in children with asthma

**DOI:** 10.1186/1476-7961-6-6

**Published:** 2008-07-31

**Authors:** Michael Pistiner, Gary M Hunninghake, Manuel E Soto-Quiros, Lydiana Avila, Amy Murphy, Jessica Lasky-Su, Brooke Schuemann, Barbara J Klanderman, Benjamin A Raby, Juan C Celedón

**Affiliations:** 1 Channing Laboratory, Department of Medicine, Brigham and Women's Hospital, Boston, MA, USA; 2 Division of Pulmonary/Critical Care Medicine, Department of Medicine, Brigham and Women's Hospital, Boston, MA, USA; 3 Division of Immunology, Children's Hospital, Boston, MA, USA; 4 Harvard Medical School, Boston, MA, USA; 5 Division of Pediatric Pulmonology, Hospital Nacional de Niños, San José, Costa Rica; 6 Dept. of Biostatistics, Harvard School of Public Health, Boston, MA, USA

## Abstract

**Background:**

IL12A has been implicated in T-cell development and may thus influence the development of atopy and allergic diseases.

**Methods:**

We tested for association between four linkage disequilibrium (LD)-tagging SNPs (rs2243123, rs2243151, rs668998, and rs17826053) in *IL12A *and asthma and allergy-related (serum total and allergen-specific IgE, and skin test reactivity [STR] to two common allergens) phenotypes in two samples: 417 Costa Rican children with asthma and their parents, and 470 families of 503 white children in the Childhood Asthma Management Program (CAMP). The analysis was conducted using the family-based association test (FBAT) statistic implemented in the PBAT program.

**Results:**

Among Costa Rican children with asthma, homozygosity for the minor allele of each of two SNPs in *IL12A *(rs2243123 and rs2243151) was associated with increased risks of STR to American cockroach (P ≤ 0.03 for both SNPs), STR to German cockroach (P ≤ 0.01 for both SNPs), and having a positive IgE to German cockroach (P < 0.05 for both SNPs). Among children in CAMP, homozygosity for the minor allele of SNP rs2243151 in *IL12A *was inversely associated with STR to German cockroach (P = 0.03) and homozygosity for the minor allele of SNP rs17826053 in *IL12A *was associated with increased risks of STR to American cockroach (P = 0.01) and STR to German cockroach (P = 0.007). There was no significant association between any SNP in *IL12A *and asthma, STR to dust mite, or total IgE in Costa Rica or CAMP.

**Conclusion:**

Our findings suggest that variants in *IL12A *influence cockroach allergy among children with asthma.

## Introduction

Interleukin 12 (IL12), an immunomodulatory cytokine secreted by antigen presenting cells, is critical for differentiation of T helper (Th)1 and Th2 lymphocytes. [1,2]. IL12 has been shown to augment the growth of activated T- and natural killer (NK)-cells [3,4], stimulate interferon gamma (IFN-γ) production by T-cells and NK cells[4,5], and suppress the expansion of Th2 cell clones [4,6].

IL12 may be implicated in the pathogenesis of asthma. Expression of IL12 is lower in airway biopsies and peripheral blood eosinophils of asthmatics than in controls [4,7]. Similarly, production of IL12 and IL12-induced release of IFN-γ are reduced in subjects with atopic asthma compared to controls [4,8]. IL12 is a disulfide-linked heterodimer comprised of IL12B (p40) and IL12A (p35) [2,9]. mRNAs for p40 and p35 are both induced upon activation and their co-expression is necessary for secretion of biologically activated IL12 [1,2].

The gene for IL12A (*IL12A*) is located on chromosome 3p12-13.2 [10], a genomic region linked to asthma and its intermediate phenotypes [11]. To date, there has been no association study of *IL12A *and asthma or allergies. Thus, we performed a study of association between variants in *IL12A *and asthma and allergy-related phenotypes in families of children with asthma in an ongoing study of the Genetics of Asthma in Costa Rica. We then attempted to replicate positive findings in Costa Rica in families of white children with asthma in the Childhood Asthma Management Program (CAMP).

## Subjects and methods

### Study populations

Subject recruitment for the Genetics of Asthma in Costa Rica Study has been previously described in detail [12]. The population of the Central Valley of Costa Rica is a genetic isolate of mixed Spanish and Amerindian ancestry [13] with a prevalence of asthma that ranks among the highest in the world [14]. In brief, Costa Rican schoolchildren aged 6–14 years were recruited from February of 2001 to March of 2005. Index children were eligible for inclusion in the study (along with their parents) if they had asthma (defined as physician-diagnosed asthma and at least 2 respiratory symptoms or asthma attacks in the previous year) and high probability of having at least 6 great-grandparents born in the Central Valley of Costa Rica [12,15]. Of the 439 participating children, 426 had DNA that passed quality control and are included in this analysis along with their parents. Index children completed a protocol that included a questionnaire (slightly modified from one used for the Collaborative Study on the Genetics of Asthma) [16], allergy skin testing, and collection of blood samples (for DNA extraction and measurement of serum total and allergen-specific IgE). Written parental consent was obtained for participating children, for whom written assent was also obtained. The study was approved by the Institutional Review Boards of the Hospital Nacional de Niños (San José, Costa Rica) and Brigham and Women's Hospital (BWH, Boston, Massachusetts).

Subject recruitment and collection of phenotypic data for CAMP have been previously described in detail [17,18]. CAMP was a multicenter clinical trial of the effects of anti-inflammatory medications in children with mild to moderate asthma. Participating children had asthma defined by symptoms greater than 2 times per week, use of an inhaled bronchodilator at least twice weekly or use of daily medication for asthma, and increased airway responsiveness to methacholine (PC_20 _≤ 12.5 mg/ml) [17,18]. Of the 1,041 children enrolled in the original clinical trial, 968 children and 1,518 of their parents contributed DNA samples. Because of small sample size for other ethnic groups, this analysis was restricted to 483 nuclear families of white children. Questionnaire data was collected at baseline and during the course of the four-year clinical trial, and blood samples and house dust samples were collected at baseline [17,18]. Written informed consent was obtained from parents of participating children. CAMP was approved by the Institutional Review Boards of BWH and the other participating centers.

#### Allergy Skin Testing

In Costa Rica, allergy skin testing was performed according to the ISAAC protocol [19]. In addition to histamine and saline controls, the following antigens were applied to the volar surface of the forearm: *Dermatophagoides (D.) pteronyssinus, D. farinae, Blatella (B.) germanica *(German cockroach), *Periplaneta (P.) americana *(American cockroach), cat dander, dog dander, mixed grass pollen, mixed tree pollen, and *Alternaria tenuis*. In CAMP, histamine, saline control, and the following allergens were applied to the volar surface of the forearm: *P. americana*, *B. germanica*, *D. pteronyssinus*, *D. farinae*, cat dander, dog dander, penicillium mix, aspergillus mix, Timothy grass, and short ragweed [17]. In Costa Rica and in CAMP, a test was considered positive if the maximum diameter of the wheal was ≥ 3 mm after subtraction of the maximum diameter of the negative control. Because of considerations of statistical power (given the known prevalence of skin test reactivity [STR] to each of the allergens tested in Costa Rica and CAMP), this analysis included only data for STR to American cockroach, STR to German cockroach, and STR to *D. pteronyssinus*.

#### Measurement of Serum Total and Allergen-Specific IgE

In Costa Rica, serum total IgE and IgE specific to two common allergens (*D. pteronyssinus *[heretofore called dust mite] and *B. germanica *[German cockroach]) were measured using the UniCAP 250 system (Pharmacia & Upjohn, Kalamazoo, MI), with samples measured in duplicate. IgE specific to each allergen was considered positive if >= 0.35 IU/ml. In CAMP, serum total IgE was measured by radioimmunosorbent assays during the screening sessions. Serum allergen-specific IgE was not measured in CAMP. In both Costa Rica and CAMP, serum total IgE was transformed to a log10 scale for data analysis.

### Genotypic data

In Costa Rica and CAMP, genotyped markers were selected using a linkage disequilibrium (LD)-tagging algorithm for *IL12A *and its 5-kb flanks. Of the 5 SNPs selected, 4 were polymorphic in Costa Rica and were successfully genotyped; these 4 SNPs capture ≥ 93% of the HapMap SNPs in *IL12A *in CEPH (Centre d'etude du polymorphisme humain) trios at an r^2 ^≥ 0.8 for a minor allele frequency (MAF) of 0.1.

In Costa Rica and CAMP, SNPs were genotyped using an Illumina Beadstation 500G system (San Diego, CA) or the Sequenom MassArray system (San Diego, CA). Duplicate genotyping was performed on approximately 5% of the sample to assess genotype reproducibility. No discordancies were found for any of the assays. All loci had > 98% genotyping completion rate.

### Statistical analysis

Hardy-Weinberg equilibrium (HWE) was tested in parental data by using a χ^2 ^goodness-of-fit test, and deviations from Mendelian inheritance were tested with PedCheck [20]. Genotypes of families with Mendelian inconsistencies were set to missing. Estimates of D' and R^2 ^were obtained from Haploview v3.11 [21].

All analyses were performed assuming additive, dominant, and recessive genetic models. In both cohorts, SNPs and haplotypes were tested for association with asthma and allergy-related phenotypes using the family-based association test statistic implemented in PBAT version v3.2 [22]. Consistent with our previous work, all analyses of quantitative phenotypes were adjusted for age and gender. Results were considered replicated at the locus level if P < 0.05 for the same phenotype under the same genetic model in both Costa Rica and CAMP.

## Results

Of 426 Costa Rican families, 9 were removed because of Mendelian inconsistencies, leaving 417 children and their parents. Of 483 nuclear families of white children in CAMP, 13 were removed because of Mendelian inconsistencies, leaving 470 families (and 503 children). All SNPs were HWE in parental genotypes in Costa Rica and CAMP. Table 1 demonstrates the baseline characteristics of index children with asthma in Costa Rica and index white (non-Hispanic) children with asthma in CAMP. The distribution of age, gender, and serum total IgE was similar in the Costa Rican and CAMP populations. However, estimates of the prevalence of STR to either American or German cockroach and the prevalence of STR to dust mite were higher in Costa Rica than in CAMP.

**Table 1:** Baseline Characteristics of Children with Asthma in Costa Rica and White Children with Asthma in CAMP

**Variable**	**Costa Rica**	**CAMP**
	N = 417	N = 503
Male gender (n, %)	260 (62.4)	312 (62.0)
Age in years (median, interquartile range)	8.7 (7.7–10.4)	8.6 (7.0–10.5)
STR to *B. germanica *(n, %)	220 (53.0)	130 (25.8)
STR to *P. americana *(n, %)	228 (54.9)	108 (21.5)
STR to *D. pteronyssinus *(%)	326 (79.0)	226 (45.0)
Positive IgE to *B. germanica *(n, %)	174 (41.7)	
Positive IgE to *D. pteronyssinus *(n, %)	319 (76.5)	
Total serum IgE, IU/ml (median, interquartile range)	414 (117–962)	399 (159–1066)

STR = skin test reactivity.

In Costa Rica, a positive IgE to either *B. germanica *or *D. pteronyssinus *was defined as a value >= 0.35 IU/ml. Allergen-specific IgE was not measured in CAMP.

**Figure 1:**
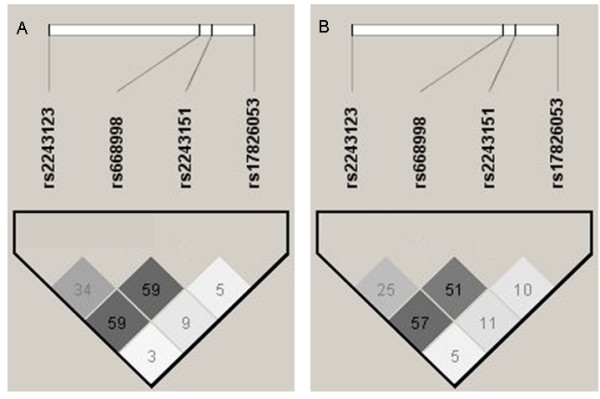
**Pairwise (r^2^) Linkage Disequilibrium Plots for *IL12A *in Parents of Index Children in A) Costa Rica and B) CAMP**.

The MAFs and the LD patterns for the SNPs of interest in *IL12A *were similar in the Costa Rican and CAMP populations (Table 2 and Figure 1)[23], and not significantly different from the MAF of these SNPs in CEPH trios (Centre d'etude du polymorphisme humain -a population of northern and western European ancestry in Utah).

**Table 2:** Allelic Frequencies of Genotyped Polymorphisms in *IL12A*

SNP (dbSNP reference number)	Minor allele	Minor allele frequency	
		Costa Rica	CAMP
rs2243123	C	0.27	0.27
rs2243151	T	0.39	0.39
rs668998	G	0.49	0.42
rs17826053	G	0.08	0.14

Table 3 shows the results of the family-based analysis of association between SNPs in *IL12A *and: a) STR to the two allergens tested (American cockroach and German cockroach) in Costa Rica and CAMP, and b) a positive IgE to German cockroach in Costa Rica (as this trait was not measured in CAMP). Among Costa Rican children with asthma, homozygosity for the minor allele of each of two SNPs (rs2243123 and rs2243151) was associated with increased risks of STR to American cockroach, STR to German cockroach, and a positive IgE to German cockroach (consistent with a recessive genetic model). Similar results were obtained under an additive genetic model, with the exception of the association between the minor allele of SNP rs2243123 and STR to German cockroach. Among children in CAMP, homozygosity for the minor allele of SNP rs2243151 was associated with reduced risk of STR to German cockroach but homozygosity for the minor allele of SNP rs17826053 was associated with increased risks of STR to American cockroach and STR to German cockroach. Although there was no association between SNP rs17826053 and STR to either American or German cockroach in Costa Rica, the frequency of the minor allele of this SNP was lower in Costa Rica (0.08) than in CAMP (0.14, see Table 2). Results of the family-based analysis of haplotypes within *IL12A *were consistent with and did not provide additional information to that gained by the analysis of SNPs (data not shown).

**Table 3:** Family-based Analysis of Association between *IL12A *and Cockroach Sensitization among Children with Asthma in Costa Rica and White Children with Asthma in CAMP

				Costa Ricans			CAMP		
SNP (dbSNP number)	Chromosome 3 position	Alleles	Location	N*	Model	FBAT P value	N*	Model	FBAT P value
**Positive IgE to *B. germanica***									
rs2243123	161192344	T>C	Intron	245	a	**+0.046**			
rs2243151	161198714	A>T	3'UTR	277	a	0.05			
				133	r	**+0.04**			
**STR to *P. Americana***									
rs2243123	161192344	T>C	Intron	244	a	**+0.03**	301	a	-
				84	r	**+0.002**	94	r	-
rs2243151	161198714	A>T	3'UTR	276	a	**+0.03**	335	a	-
				133	r	**+0.01**	169	r	-
rs17826053	161200322	T>G	3'UTR	120	a	-	203	a	**+0.02**
				119	r	-	198	r	**+0.01**
**STR to B. germanica**									
rs2243123	161192344	T>C	Intron	244	a	-	301	a	-
				84	r	**+0.01**	94	r	0.09
rs2243151	161198714	A>T	3'UTR	276	a	0.08	335	a	0.09
				133	r	**+0.0007**	169	r	**-0.03**
rs17826053	161200322	T>G	3'UTR	120	a	-	203	a	**+0.004**
				119	r	-	198	r	**+0.007**

*Number of informative families.

STR = skin test reactivity.

+ and - signs preceding significant P values indicate the direction of the observed associations.

In Costa Rica, a positive IgE to *B. germanica *was defined as a value >= 0.35 IU/ml. Allergen-specific IgE was not measured in CAMP.

3' UTR = 3' untranslated region.

For ease of exposition, only p values < 0.1 are displayed. A dash "-" has been used to represent p values ≥ 0.1.

There was no significant association between any of the SNPs in *IL12A *and asthma, total IgE, STR to dust mite, or IgE to dust mite (Table 4).

**Table 4:** Family-based Analysis of Association between *IL12A *and Asthma, Total IgE, and STR to Dust Mite among Children with Asthma in Costa Rica and CAMP

SNP (dbSNP rs number)	Chromosome 3 position	Alleles	Location	Costa Ricans			Whites		
				N*	Model	FBAT p-value	N*	Model	FBAT p-value
**Asthma**									
rs2243123	161192344	T>C	Intron	245	a	0.86	301	a	0.58
rs2243151	161198714	A>T	3'UTR	277	a	0.46	335	a	0.49
rs668998	161198244	A>G	3'UTR	282	a	0.96	337	a	0.36
rs17826053	161200322	T>G	3'UTR	121	a	0.42	203	a	0.14
**Total IgE**									
rs2243123	161192344	T>C	Intron	245	a	0.30	301	a	0.54
rs2243151	161198714	A>T	3'UTR	277	a	0.41	335	a	0.60
rs668998	161198244	A>G	3'UTR	282	a	0.32	337	a	0.47
rs17826053	161200322	T>G	3'UTR	121	a	0.61	203	a	0.19
**Positive IgE to *D. pteronyssinus***									
rs2243123	161192344	T>C	Intron	245	a	0.30			
rs2243151	161198714	A>T	3'UTR	277	a	0.43			
rs668998	161198244	A>G	3'UTR	282	a	0.67			
rs17826053	161200322	T>G	3'UTR	121	a	0.60			
**STR to *D. pteronyssinus***									
rs2243123	161192344	T>C	Intron	244	a	0.95	301	a	0.21
rs2243151	161198714	A>T	3'UTR	276	a	0.77	335	a	0.18
rs668998	161198244	A>G	3'UTR	280	a	0.52	337	a	0.10
rs17826053	161200322	T>G	3'UTR	120	a	0.43	203	a	0.85

*Number of informative families

In Costa Rica, a positive IgE to *D. pteronyssinus *was defined as a value >= 0.35 IU/ml.

Allergen-specific IgE was not measured in CAMP.

## Discussion

Among children with asthma in two ethnically distinct cohorts, SNPs in *IL12A *were associated with an increased risk of sensitization to cockroach but not with asthma or other objective markers of atopy (serum total IgE, STR or IgE to dust mite). The association between *IL12A *polymorphisms and cockroach allergy but not asthma is consistent with results from human studies of recombinant IL12 [24].

To our knowledge, this is the first study to examine the association between polymorphisms in *IL12 A *and asthma and allergy-related phenotypes. Strengths of this study include its family-based design (which eliminates concerns for population stratification) and the relatively large sample size and availability of objectively measured allergy-related phenotypes for each of the two cohorts included.

We have previously demonstrated that the genomic regions that influence sensitization to cockroach differ from those that influence sensitization to dust mite in Costa Rica [25]. Thus, our findings for *IL12A *may be due to a more marked influence of variants in this gene on cockroach sensitization than on other allergy phenotypes among children with asthma. Prior genetic predisposition to cockroach sensitization has been previously demonstrated for variants in the genes for HLA-DRB1*01 and HLA-DRB1*02 in Hutterite and African-American populations [26]. Of note, sensitization to cockroach has been shown to be associated with disease severity among children with asthma, particularly in the presence of high levels of cockroach allergen [27].

As with any genetic association study, type I (false positive) and type II (false negative) results should be considered. With regard to potential type I error, the observed association between variants in *IL12A *and cockroach sensitization (assessed in two different ways in Costa Rica [measurement of specific IgE and STR]) was consistent at the gene (locus) level in the two populations studied. At the SNP level, the observed association between the minor allele of SNP rs2243151 and cockroach sensitization in Costa Rica would remain significant even after an stringent Bonferroni correction for multiple testing (P < 0.001 or 0.05/48 [3 (number of genetic models] * 4 (number of SNPs tested) * 4 (number of distinct phenotypes, given the correlation between measures of both cockroach and dust mite sensitization) under a recessive genetic model. The consistency of the positive association between SNP rs2243151 and two measures of cockroach sensitization in the same genetic model in Costa Rica also decreases the likelihood that this is a false positive association. With regard to potential type II error, differences in the prevalence of sensitization to cockroach between study populations may have resulted in reduced power to detect associations in CAMP and thus lack of replication for some of our findings in Costa Rica. Conversely, the discrepant findings for SNP rs17826053 may reflect reduced statistical power due to a low minor allele frequency for this variant in Costa Rica.

LD with variants in adjacent genes is an unlikely explanation for our findings because the closest gene on either flank of *IL12A *(schwannomin interacting protein 1 [*SCHIP1*]) is located ~100 kb away. On the other hand, LD with other (non-genotyped) variants in *IL12A *could partly explain our results. To begin to examine this question, we attempted to genotype a coding SNP in *IL12A *that has been validated in dbSNP build 129 (rs1042155). However, this variant was monomorphic in Costa Rica.

While the observed association between SNP rs2243151 and sensitization to German cockroach was significant in both cohorts under a recessive genetic model, it was not in the same direction across samples (positive in Costa Rica, negative or inverse in CAMP). Given that at least one other SNP was associated with an increased risk of STR to cockroach in CAMP, the "flip-flop" association for SNP rs2243151 may be due to subtle differences in LD patterns for *IL12A *or unmeasured gene-by-gene or gene-by-environment interactions in Costa Rica and/or CAMP [28]. Because of the small number of children with significant exposure to cockroach allergen in the Central Valley of Costa Rica, we had very limited power to assess gene-by-cockroach allergen interactions.

In summary, this study demonstrates that polymorphisms in *IL12A *are associated with sensitization to cockroach among children with asthma. Definitive identification of the functional SNPs responsible for this association will require further study in our cohorts and in other populations.

## Abbreviations

B. Germanica: Blatella germanica; CAMP: Childhood Asthma Management Program; D. pteronyssinus: Dermatophagoides pteronyssinus; IgE: Immunoglobulin E; IFN-γ: Interferon gamma; IL12: Interleukin 12;  ISAAC: International Study of Asthma and Allergies in Childhood; NK: Natural killer; P. americana: Periplaneta americana; SNP: Single Nucleotide Polymorphism; Th: T helper; STR: Skin Test Reactivity. 

## Competing interests

MP, GMH, MESQ, LA, AM, JS, BS, BJK, BAR, and JCC do not have a financial relationship with a commercial entity that has an interest in the subject of this manuscript.

## Authors' contributions

MP, GMH, and BAR declare that they have participated in the data analysis and statistical support for this manuscript. MESQ, LA, BS, and BJK have participated in the funding and data collection for this manuscript. AM, and JLS declare that they have participated in the statistical support for this manuscript. JCC declares that he participated in the funding, study design, data collection, data analysis, and statistical support. All authors have participated in manuscript writing/editing and have seen and approved the final version of this manuscript.

## References

[B1] Szabo SJ, Jacobson NG, Dighe AS, Gubler U, Murphy KM (1995). Developmental commitment to the Th2 lineage by extinction of IL-12 signaling. Immunity.

[B2] Wills-Karp M (2001). IL-12/IL-13 axis in allergic asthma. J Allergy Clin Immunol.

[B3] Robertson MJ, Soiffer RJ, Wolf SF, Manley TJ, Donahue C, Young D, Herrmann SH, Ritz J (1992). Response of human natural killer (NK) cells to NK cell stimulatory factor (NKSF): cytolytic activity and proliferation of NK cells are differentially regulated by NKSF. J Exp Med.

[B4] Chung F (2001). Anti-inflammatory cytokines in asthma and allergy: interleukin-10, interleukin-12, interferon-gamma. Mediators Inflamm.

[B5] Kobayashi M, Fitz L, Ryan M, Hewick RM, Clark SC, Chan S, Loudon R, Sherman F, Perussia B, Trinchieri G (1989). Identification and purification of natural killer cell stimulatory factor (NKSF), a cytokine with multiple biologic effects on human lymphocytes. J Exp Med.

[B6] Manetti R, Parronchi P, Giudizi MG, Piccinni MP, Maggi E, Trinchieri G, Romagnani S (1993). Natural killer cell stimulatory factor (interleukin 12 [IL-12]) induces T helper type 1 (Th1)-specific immune responses and inhibits the development of IL-4-producing Th cells. J Exp Med.

[B7] Naseer T, Minshall EM, Leung DY, Laberge S, Ernst P, Martin RJ, Hamid Q (1997). Expression of IL-12 and IL-13 mRNA in asthma and their modulation in response to steroid therapy. Am J Respir Crit Care Med.

[B8] Pouw Kraan TC van der, Boeije LC, de Groot ER, Stapel SO, Snijders A, Kapsenberg ML, Zee JS van der, Aarden LA (1997). Reduced production of IL-12 and IL-12-dependent IFN-gamma release in patients with allergic asthma. J Immunol.

[B9] Trinchieri G (1994). Interleukin-12: a cytokine produced by antigen-presenting cells with immunoregulatory functions in the generation of T-helper cells type 1 and cytotoxic lymphocytes. Blood.

[B10] Sieburth D, Jabs EW, Warrington JA, Li X, Lasota J, LaForgia S, Kelleher K, Huebner K, Wasmuth JJ, Wolf SF (1992). Assignment of genes encoding a unique cytokine (IL12) composed of two unrelated subunits to chromosomes 3 and 5. Genomics.

[B11] Meyers DA, Postma DS, Stine OC, Koppelman GH, Ampleford EJ, Jongepier H, Howard TD, Bleecker ER (2005). Genome screen for asthma and bronchial hyperresponsiveness: interactions with passive smoke exposure. J Allergy Clin Immunol.

[B12] Hunninghake GM, Soto-Quiros ME, Avila L, Ly NP, Liang C, Sylvia JS, Klanderman BJ, Silverman EK, Celedon JC (2007). Sensitization to Ascaris lumbricoides and severity of childhood asthma in Costa Rica. J Allergy Clin Immunol.

[B13] Carvajal-Carmona LG, Ophoff R, Service S, Hartiala J, Molina J, Leon P, Ospina J, Bedoya G, Freimer N, Ruiz-Linares A (2003). Genetic demography of Antioquia (Colombia) and the Central Valley of Costa Rica. Hum Genet.

[B14] Pearce N, Ait-Khaled N, Beasley R, Mallol J, Keil U, Mitchell E, Robertson C (2007). Worldwide trends in the prevalence of asthma symptoms: phase III of the International Study of Asthma and Allergies in Childhood (ISAAC). Thorax.

[B15] Freimer NB, Reus VI, Escamilla M, Spesny M, Smith L, Service S, Gallegos A, Meza L, Batki S, Vinogradov S (1996). An approach to investigating linkage for bipolar disorder using large Costa Rican pedigrees. Am J Med Genet.

[B16] Blumenthal MN, Banks-Schlegel S, Bleecker ER, Marsh DG, Ober C (1995). Collaborative studies on the genetics of asthma – National Heart, Lung and Blood Institute. Clin Exp Allergy.

[B17] (1999). The Childhood Asthma Management Program (CAMP): design, rationale, and methods. Childhood Asthma Management Program Research Group. Control Clin Trials.

[B18] (2000). Long-term effects of budesonide or nedocromil in children with asthma. The Childhood Asthma Management Program Research Group. N Engl J Med.

[B19] Weiland SK, Bjorksten B, Brunekreef B, Cookson WO, von Mutius E, Strachan DP (2004). Phase II of the International Study of Asthma and Allergies in Childhood (ISAAC II): rationale and methods. Eur Respir J.

[B20] O'Connell JR, Weeks DE (1998). PedCheck: a program for identification of genotype incompatibilities in linkage analysis. Am J Hum Genet.

[B21] Barrett JC, Fry B, Maller J, Daly MJ (2005). Haploview: analysis and visualization of LD and haplotype maps. Bioinformatics.

[B22] Laird NM, Horvath S, Xu X (2000). Implementing a unified approach to family-based tests of association. Genet Epidemiol.

[B23] Hunninghake GM, Soto-Quiros ME, Avila L, Su J, Murphy A, Demeo DL, Ly NP, Liang C, Sylvia JS, Klanderman BJ (2007). Polymorphisms in IL13, total IgE, eosinophilia, and asthma exacerbations in childhood. J Allergy Clin Immunol.

[B24] Bryan SA, O'Connor BJ, Matti S, Leckie MJ, Kanabar V, Khan J, Warrington SJ, Renzetti L, Rames A, Bock JA (2000). Effects of recombinant human interleukin-12 on eosinophils, airway hyper-responsiveness, and the late asthmatic response. Lancet.

[B25] Hunninghake GM, Lasky-Su J, Soto-Quiros ME, Avila L, Liang C, Lake SL, Hudson TJ, Spesny M, Fournier E, Sylvia JS (2008). Sex-stratified linkage analysis identifies a female-specific locus for IgE to cockroach in Costa Ricans. Am J Respir Crit Care Med.

[B26] Donfack J, Tsalenko A, Hoki DM, Parry R, Solway J, Lester LA, Ober C (2000). HLA-DRB1*01 alleles are associated with sensitization to cockroach allergens. J Allergy Clin Immunol.

[B27] Rosenstreich DL, Eggleston P, Kattan M, Baker D, Slavin RG, Gergen P, Mitchell H, McNiff-Mortimer K, Lynn H, Ownby D, Malveaux F (1997). The role of cockroach allergy and exposure to cockroach allergen in causing morbidity among inner-city children with asthma. N Engl J Med.

[B28] Lin PI, Vance JM, Pericak-Vance MA, Martin ER (2007). No gene is an island: the flip-flop phenomenon. Am J Hum Genet.

